# Sex and age modulate the relationship between melanopsin-dependent light sensitivity and chronotype

**DOI:** 10.1093/sleep/zsag057

**Published:** 2026-02-27

**Authors:** Gijs van der Zwet, Zoë Bor, Roos Bos, Rick van Dorp, Laura M Pape, Laura C A van der Zwet, Elon H C van Dijk, Huybert van de Stadt, Elise M McGlashan, Stephan Michel, Laura Kervezee

**Affiliations:** Group of Circadian Medicine, Department of Cell and Chemical Biology, Leiden University Medical Center, Einthovenweg 20, Leiden, ZC 2333, The Netherlands; Group of Circadian Medicine, Department of Cell and Chemical Biology, Leiden University Medical Center, Einthovenweg 20, Leiden, ZC 2333, The Netherlands; Group of Circadian Medicine, Department of Cell and Chemical Biology, Leiden University Medical Center, Einthovenweg 20, Leiden, ZC 2333, The Netherlands; Department of Neonatology, Wilhelmina Children’s Hospital, University Medical Center Utrecht, Heidelberglaan 100, Utrecht, CX 3584, The Netherlands; Group of Neurophysiology, Department of Cell and Chemical Biology, Leiden University Medical Center, Einthovenweg 20, Leiden, ZC 2333, The Netherlands; Department of Clinical Psychology, Faculty of Social and Behavioural Sciences, Leiden University, Wassenaarseweg 52, Leiden, AK 2333, The Netherlands; Group of Neurophysiology, Department of Cell and Chemical Biology, Leiden University Medical Center, Einthovenweg 20, Leiden, ZC 2333, The Netherlands; Department of Ophthalmology, Leiden University Medical Center, Albinusdreef 2, Leiden, ZA 2333, The Netherlands; Department of Vitreoretinal Surgery, Rotterdam Eye Hospital, Rotterdam, The Netherlands; Department of Medical Technology, Leiden University Medical Center, Einthovenweg 20, Leiden, ZC 2333, The Netherlands; Melbourne School of Psychological Sciences, University of Melbourne, Parkville, VIC 3010, Australia; Group of Circadian Medicine, Department of Cell and Chemical Biology, Leiden University Medical Center, Einthovenweg 20, Leiden, ZC 2333, The Netherlands; Group of Neurophysiology, Department of Cell and Chemical Biology, Leiden University Medical Center, Einthovenweg 20, Leiden, ZC 2333, The Netherlands; Group of Circadian Medicine, Department of Cell and Chemical Biology, Leiden University Medical Center, Einthovenweg 20, Leiden, ZC 2333, The Netherlands

**Keywords:** non-visual effects of light, circadian rhythms, light, chronotype, melanopsin, PIPR, post-illumination pupillary response

## Abstract

**Study Objectives:**

Light, acting primarily via melanopsin-mediated signaling, plays a central role in synchronizing circadian rhythms. Individuals vary markedly in the sensitivity of their circadian system to light. Whether these differences contribute to the interindividual variability in chronotype, a behavioral manifestation of internal circadian timing, is unclear. The aim of this study was to determine the relationship between melanopsin-dependent light sensitivity and chronotype and to assess whether age and sex modulate this association across the general population.

**Methods:**

Participants (adults and children aged ≥ 8 years) were recruited in a science museum. Chronotype was determined using the μMCTQ. The post-illumination pupillary response (PIPR) was used as a measure of melanopsin-dependent light sensitivity. The relationship between PIPR and chronotype and their interaction with age and sex were assessed using multiple linear regression.

**Results:**

Pupil recordings and questionnaires were obtained from 433 participants, including 269 adults (age range: 18-75 years) and 164 children (age range: 8-17 years). In adults, the relationship between melanopsin-dependent light sensitivity and chronotype depends on sex and age: greater light sensitivity is linked to a significantly later chronotype in young adult men and to an earlier chronotype in older adult women. In children, no evidence was found for a relationship between light sensitivity and chronotype.

**Conclusion:**

Individual variation in light sensitivity interacts with sex- and age-specific differences in the circadian system and light exposure behavior to influence circadian timing. Light exposure recommendations should be personalized to take into account these sex- and age-specific effects.

## Graphical Abstract

**Graphical Abstract:**
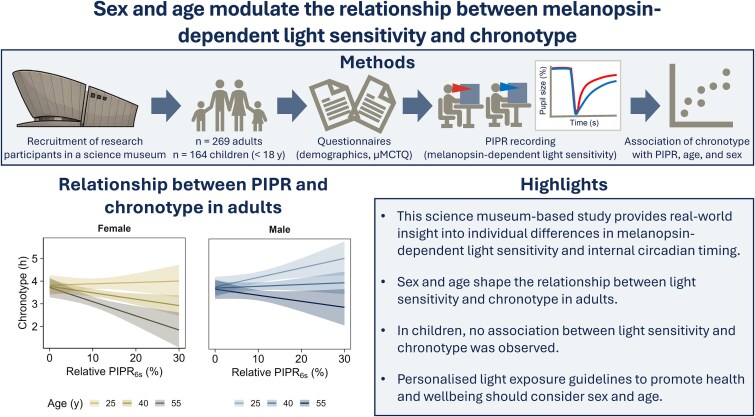

Statement of Significance While individuals differ widely in how their circadian system responds to light, to what extent these individual responses influence internal circadian timing remains unclear. By studying a large and diverse sample of children and adults, our findings offer a more comprehensive understanding of the relationship between light sensitivity and chronotype than previously recognized. Sex and age shape this relationship: greater light sensitivity is linked to later chronotype in young men but to earlier chronotype in older women. These results reveal that the impact of light on circadian timing changes across the lifespan and differs by sex. As such, this study contributes to the development of personalized light exposure guidelines to promote health and well-being.

## Introduction

Individuals differ substantially in the timing of their internal circadian rhythms, which can manifest in daily life as differences in chronotype, ranging from early chronotypes (“early birds”) to late chronotypes (“night owls”). Chronotype is influenced by genetic factors, sex, as well as age [1–3] and is linked to a number of lifestyle factors and health outcomes, with later chronotypes showing a higher prevalence of alcohol intake and tobacco use [4], adverse metabolic outcomes, and depressive symptoms [5]. Therefore, characterizing the biological and behavioral factors that underlie the variation in chronotype across the population is important for the development of strategies that promote sleep and circadian health at the population level.

Another factor that may contribute to the variation in chronotype is interindividual differences in the response to environmental timing cues that synchronize the circadian system to the external environment, such as light [6, 7]. Individuals vastly differ in how sensitive their circadian system is to light [8], which may lead to differences in circadian timing [9]. In addition, interindividual differences in habitual light exposure patterns may further amplify variations in circadian timing, because the response of the circadian timing system to light depends on time of day, with light exposure in the morning leading to phase advances and in the evening to phase delays. Importantly, since both age and sex influence circadian biology, light sensitivity, as well as habitual light exposure patterns [6, 10, 11], these characteristics may further modulate the association between circadian timing and light sensitivity.

Light is conveyed to the central circadian clock in the suprachiasmatic nucleus of the hypothalamus via intrinsically photosensitive retinal ganglion cells (ipRGCs) in the eye [12–14]. IpRGCs contain the photopigment melanopsin that is sensitive to short wavelengths of visible light (i.e. blue light) [15–17]. Besides synchronizing the circadian system to the environment, melanopsin-mediated phototransduction via ipRGCs is known to mediate other non-visual effects of light [18], such as the pupillary light response, via direct projections to the olivary pretectal nucleus [12]. Specifically, ipRGCs drive the post-illumination pupillary response (PIPR), the sustained constriction of the pupil after light offset, providing a non-invasive measure for assessing individual differences in melanopsin-mediated phototransduction.

Prior studies in specific cohorts provide some evidence for increased melanopsin-mediated pupil responses being associated with later sleep or circadian timing. For example, in a cohort of young healthy adults, a more sustained PIPR was associated with later sleep timing [19], and in a cohort of adults with varying depression severity, a more sustained PIPR was associated with later circadian timing in summer but not in winter [20]. Additionally, individuals with delayed sleep–wake disorder (sometimes conceptualized as an extremely late chronotype) may show altered pupillary responses to light that contribute to differences in sleep and circadian timing [21, 22]. However, it remains unclear to what extent variation in melanopsin-mediated phototransduction can explain differences in chronotype across the general population and whether this association is influenced by age and sex. Insight into these relationships is essential to advance our understanding of the biological basis of chronotype and to develop effective, personalized, light exposure recommendations.

Therefore, to further characterize the relationship between melanopsin-mediated phototransduction and chronotype in the general population, the aim of this study was to investigate this association across a wide age range and in different sexes. Making use of the unique opportunity to conduct this study in a sample of science museum visitors, our goal was not only to elucidate the role of melanopsin-mediated phototransduction in shaping individual chronotype but also to promote awareness of circadian biology in a broad community, thereby bridging the gap between science and society.

## Materials and Methods

### Participants

This study was conducted as part of the Science Live program of the NEMO Science Museum (Amsterdam, the Netherlands). Participants were recruited and studied during museum opening hours (10:00-17:30) on an ongoing basis during a two-week school holiday (December 21, 2024–January 5, 2025). On December 27, 2024, the middle of the study period, the photoperiod (daylight hours) was 7 hours and 42 minutes in Amsterdam, with sunrise at 08:50 and sunset at 16:32. Visitors of the museum, who were ≥ 8 years old and proficient in Dutch or English, were invited to participate in this study as part of their visit to the museum. Up to four participants could be accommodated per session allowing for groups of visitors (e.g. families) to participate together. Children younger than 18 years were accompanied by a parent or guardian to sign informed consent. Participants were subsequently assigned a random identification number and all research data were collected anonymously. Participants were excluded from the study if they reported having epileptic disorders, using illicit drugs or cannabis in the past seven days, or using pupil-dilating eye drops in the past two weeks. In addition, they were excluded if they reported being unable or unwilling to be in a dimly lit room for ~10 minutes (e.g. due to nyctophobia or claustrophobia), or if they reported experiencing discomfort after receiving a light stimulus in the dark (e.g. due to photophobia or migraine). The study protocol was approved by the Medical Ethics Committee (nWMO Commissie Divisie 4) of the Leiden University Medical Center under registration number nWMO-D4-2024-020.

### Study protocol

After providing written informed consent and passing screening, participants were asked to fill out a questionnaire about basic demographics, general health and lifestyle (including smoking, caffeine, and alcohol intake), use of medication, sleep habits and ocular health. Chronotype was determined by the micro Munich Chronotype Questionnaire (μMCTQ) [23]. For questions in the μMCTQ related to sleep and wake timing on free days, participants were explicitly instructed to answer these questions with a day in mind that is free of commitments or disturbances, such as alarm clocks or house mates (e.g. young children) that may disrupt their sleep. A separate version of the questionnaire was used for children (participants below the age of 18), in which the questions about alcohol use, smoking, and night shift work were omitted and questions about chronotype were worded differently to be more applicable (e.g. school days instead of working days). A member of the research team was present at all times to answer questions or clarify possible misunderstandings while participants filled in the questionnaires. After filling in the questionnaires, participants were taken to a separate room to undergo the pupil recording. Prior to the start of the pupil recording, the participants were informed about each step of the experimental protocol, and the light stimuli were demonstrated to prepare participants for the brightness of the light stimuli. Subsequently, the main overhead lights were turned off and the participants underwent a dark adaptation period (< 2 photopic lux) of 5 minutes, in line with previous studies [22, 24–26].

After dark adaptation, a monocular pupil recording of the right eye and the light exposure protocol were started. For the light protocol, baseline pupil diameter data were captured for 30 seconds in the dark, followed by a 1-second pulse of red light, 60 seconds of inter-stimulus darkness, a 1-second pulse of blue light, and 30 seconds of post-stimulus darkness (Supplementary Figure S1A). The red light stimulus served as a control to account for the contribution of other retinal photoreceptors to the PIPR and was shown before the blue light stimulus to ensure that the pupil reached its baseline diameter again during the interstimulus interval. The interstimulus interval was kept as short as possible (60 seconds) and the red and blue light stimuli were presented once to limit the total experimental time, which was an important consideration in the museum setting. Participants were asked approximately 10 seconds prior to a light pulse to move and blink as little as possible until 10 seconds after light offset. After completion of the pupil recording, the room lights were turned on again and the participants were shown images of their pupil before and after the two light pulses (Supplementary Figure S1B).

### Light stimuli

Four identical experimental setups were developed that consisted of a custom-made rectangular light panel of 27 cm x 18 cm (width x height) that consisted of RGB LED array (NeoPixel WS2812, Adafruit, New York City, USA) covered by diffuser material with optimal light transmittance (PyraLed Makrolon Dx NR 139 cool, LT = 87%, Pyrasied, Leeuwarden, the Netherlands) (Supplementary Figure S1C). The light color and intensity of the light panel was controlled by an Adafruit Gemma controller (Adafruit, New York City, USA). A custom-made user interface (Python v 3.11.5) was used to control light exposures via an Arduino Uno microcontroller (Arduino, Monza, Italy) that triggered the RGB LEDs and to log the time stamps of light pulses, which was saved as a csv file after completion of the experimental protocol.

The pupil recording was performed in a dimly lit room (< 2 photopic lux). During the experiment, the distance between the participants’ cornea and the LED panel was 15 cm, yielding a visual angle of 84° x 62° (horizontal x vertical plane). The camera recorded the pupil through a cutout in the LED panel that covered approximately 20° of the central visual field. Irradiance at the level of the cornea was measured in the vertical plane for each of the experimental setups with a calibrated spectrometer (Avantes 1901283u1, Avantes, Apeldoorn, the Netherlands) using AvaSoftB software (AvaSoft 8.7.1.0–2017 Avantes) and converted to total irradiance and melanopic equivalent daylight illuminance (mEDI) using the CIE S 026 toolbox [27]. The red light had a total irradiance of 14.0 log photons/cm^2^/s, a mEDI of 1.3 lux, a photopic illuminance of 73 lux, and a peak wavelength of 627 nm with a bandwidth at half-max of 15 nm (Supplementary Figure S1D). The blue light had a total irradiance of 14.0 log photons/cm^2^/s, a mEDI of 244 lux, a photopic illuminance of 26 lux, and a peak wavelength of 463 nm with a bandwidth at half-max of 17 nm (Supplementary Figure S1D). Light conditions are reported in line with the ENLIGHT reporting guidelines [28].

### Pupil recordings

The pupil recordings were made using a custom-made setup, consisting of an infrared light source (M120, Kemo Electronic GmbH, Germany) and a monochrome camera (Basler daA1440-220um with Basler pylon 6.0.1 drivers) equipped with an infrared filter (IR850, 30.5 mm, Green.L, China). Images were acquired using open-source PupilEXT software (beta version 0.1.1) [29]. Camera calibration was performed in PupilEXT with default settings. Throughout the experimental runs, the gain was set to 8 dB and the exposure time to 9 ms, while the aperture and focus of the camera were adapted to the individual participant to optimize brightness and contrast of the recording. The camera was set to capture images at 60 frames per second. For each image, the pupil diameter and the pupil outline confidence were determined by PupilEXT using the PuReST algorithm [30]. These data, along with the timestamps of the image acquisition, were stored in a csv file and used for further analysis.

### Pupil data processing

All data processing and subsequent statistical analyses were performed in R v4.4.0 [31]. The light exposure protocol was matched to the pupil diameter timeseries using the recorded timestamps. Preprocessing of the raw pupil diameter time series was performed for each participant similar to the steps described by Martin et al. [32]. Firstly, to account for blinks, pupil diameter values were discarded if the first derivative of the diameter value exceeded ±3 times the standard deviation or if the outline confidence was below 0.8. An additional preprocessing step was incorporated to account for cases in which the iris rather than the pupil was inadvertently detected by the algorithm, by setting values to missing if they were > 20% larger than the baseline value (i.e. the median pupil diameter 5 seconds before the red and blue lights were switched on). Missing values were replaced using linear interpolation. Timeseries were subsequently smoothened using a third-order Butterworth filter with a 4 Hz cutoff [32].

Next, timeseries were subjected to a quality control process to verify the reliability of the pupil recordings. Firstly, the stability of the timeseries was summarized by computing the relative median derivative of the smoothened timeseries at baseline and during the 6-second PIPR windows (between 5 and 7 seconds after lights off). If this value exceeded 20% (indicating instability of the recording, e.g. due to alternating detection of the pupil and the iris by the algorithm), the quality of the recording was deemed insufficient and the recording was excluded from further analysis. In addition, all pupil recordings were visually inspected independently by two authors (ZB and LK), along with the notes documented at the time of the experiment. Recordings were marked for exclusion if the experimental notes indicated a lack of compliance, accidental blinking during a light pulse, or if visual inspection revealed an unstable recording. Exclusions were then decided on by consensus between the two authors.

Following preprocessing, relative pupil diameter was calculated by expressing pupil diameter following the red and blue light onset as a percentage of the median (baseline) pupil diameter in the 5 seconds prior to the red and blue light onset, respectively. Subsequently, the net PIPR_6s_ was calculated from each timeseries by subtracting the median relative pupil diameter from 5 to 7 seconds after the blue light offset from the median relative pupil diameter from 5 to 7 seconds after the red light offset [33]. Negative PIPR_6s_ values were excluded from further analysis. The PIPR_6s_ was selected because it has least intra-individual variability compared to other PIPR metrics [34] and it was considered most suitable in the museum setting as it can be captured over a shorter duration than more sustained PIPR metrics. Furthermore, despite showing circadian variation, PIPR_6s_ is minimally influenced by time of day during office hours (i.e. museum opening hours) [35–37].

### Statistical analysis

Data from adults (≥ 18 years old) and children (8–17 years old) were analyzed separately. As a first step, linear regression models were used to determine the effect of PIPR_6s_ (independent variable; continuous) on chronotype (dependent variable; continuous). Chronotype was determined using the μMCTQ by computing the midpoint of sleep on free days corrected for sleep debt (MSF_SC_) [23]. Chronotype is expressed in hours, with smaller values indicating early chronotypes (colloquially known as “morning types”) and larger values indicating late chronotypes (known as “evening types”). For all statistical analyses, main effects and interactions were considered significant if *p*<.05 (two-sided).

To assess if age and sex modulate the relationship between PIPR_6s_ and chronotype, linear regression models were used that included PIPR_6s_ (continuous variable), sex (categorical variable), and age (continuous variable) as main effects as well as their three-way and two-way interaction terms (“full model”). Continuous predictors were standardized (i.e. scaled to have a mean of 0 and a standard deviation of 1) before entry into the linear regression model. Significance of main effects and interaction terms was assessed using Type II sum of squares analysis of variance tests using the R package car (v 3.1-3) [38]. Non-significant (higher-order) interaction terms were subsequently removed from the full three-way interaction model to arrive at the final model that was used to interpret main effects and (if present) significant lower-order interaction terms. Model assumptions (linearity and normality of residuals) were examined using histograms and Q-Q plots of the residuals.

To report the (back-transformed) model-estimated slopes and 95% confidence intervals (CIs) of the relationship between the chronotype and the predictors (and the significant interaction terms), we applied the Johnson-Neyman technique using the *estimate_slopes* function from the R package modelbased (v0.13.1) [39]. The unit of the reported slopes and 95% CIs were expressed as the change in chronotype (in hours) for each 10% point change in PIPR_6s_. In addition, the R package emmeans (v1.11.1) [40] was used to visualize estimated marginal means in order to facilitate interpretation of significant main effects and significant interaction terms.

A sensitivity analysis was carried out to examine to what extent the observed relationships are present in a “clean” adult population that is more similar to study populations recruited for typical controlled laboratory studies. To this end, the final model was fit to a dataset from which all participants were excluded who reported any of the following criteria: being color blind, working a night shift more than once in the past month, taking any sleep medication in the past night, having smoked or drunk alcohol on the day of the experiment, or drinking more than one caffeinated beverage on the day of the experiment. In addition, all participants that reported having ocular abnormalities or taking any medication in the past 24 hours that may affect the study outcomes (reviewed by study physician EvD) were excluded from this analysis. Model estimates for this sensitivity analysis were visualized as described above for the main analysis.

## Results

### Participants

**Figure 1:**
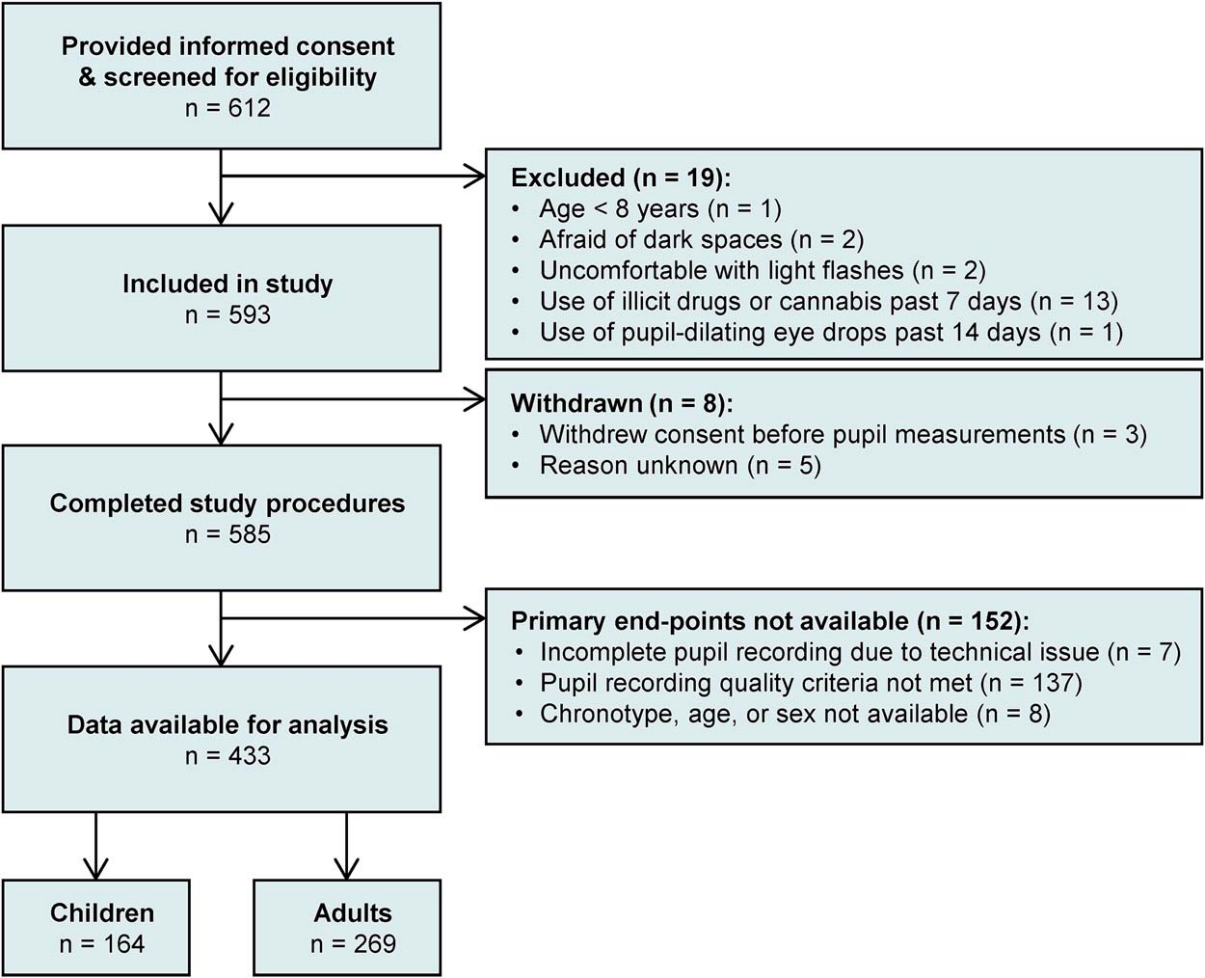
Flowchart of participant inclusion.

**Figure 2:**
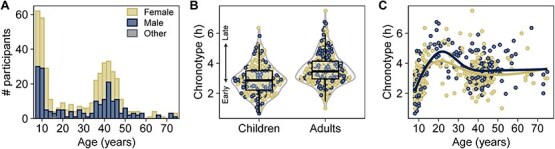
Distribution of age and chronotype and their relationship. (A) Age distribution of all 433 included participants, split in females (*n* = 238), males (*n* = 194), and others (*n* = 1). (B) Chronotype distribution in children (*n* = 164) and adults (*n* = 269). Boxplots are shown as median and interquartile ranges (IQR), with whiskers extending to 1.5 IQR. Chronotype is defined as the midpoint of sleep on free days corrected for sleep debt (MSF_SC_) in hours. (C) Relationship between age and chronotype (MSF_SC_) across sexes. Solid lines indicate smoothed curves fitted through the data for males and females separately using loess regression. Color coding in panel B and C as in panel A.

Of the total of 612 participants that provided written informed consent and underwent the screening, data from 433 participants were available for analysis following data cleaning (see Figure 1 for the inclusion flowchart), of which 269 were adults (18–75 years old) and 164 were children (8–17 years old). The total number of included participants per day ranged from 25 to 49, with a median of 32 recordings per day (Supplementary Figure S2). For detailed participant information, see Table 1. The age distribution of the entire study population was bimodal (Figure 2A), reflecting the expected demographic composition of science museum visitors during a school holiday, consisting predominantly of children and their parents. Children had an earlier chronotype (MSF_SC_: 2.86 [IQR: 2.20–3.50] h) than adults (3.46 [IQR: 2.96–4.14] h, t[351] = 6.8, *p*<.001) (Table 1, Figure 2B). Of note, we found that chronotype depends on sex and age, with chronotype being generally earlier in women than in men and becoming progressively later from childhood through adolescence, then gradually shifting to earlier in adulthood (Figure 2C), visually similar to what has been observed previously in large epidemiological studies [41–43]. Lastly, the time of day that adult participants participated in the experiment was significantly associated with chronotype (*p*=.041), with participants undergoing the pupillary assessments at 11:00 having on average a 0.43 h (95% CI: 0.017–0.85) earlier chronotype than those at 17:00 (Supplementary Figure S3, left), presumably reflecting that later chronotypes, on average, tend to visit the museum later than early chronotypes. This association was not observed in children (Supplementary Figure S3, right).

**Table 1:** Participant characteristics

Characteristic	Adults (*n* = 269)	Children (*n* = 164)
**Age (y)**	41 (36, 46)	10 (9, 12)
**Sex**		
Female	145 (54%)	93 (57%)
Male	124 (46%)	70 (43%)
Other	0 (0%)	1 (0.6%)
**Chronotype (MSF**_**sc**_ **; h)**	3.46 (2.96, 4.14)	2.86 (2.20, 3.50)
**Social jetlag (h)**	0.75 (0.50, 1.38)	1.00 (0.48, 1.67)
**Trouble sleeping prior night**	57 (21%)	39 (24%)
**Night shift work**	17 (6.3%)	ND
**Smoking on experimental day**	12 (4.5%)^*^	ND
**Alcohol consumption on experimental day**	0 (0%)^*^	ND
**Caffeine intake on experimental day (number of cups)**		
0	61 (24%)	147 (90%)
1	85 (33%)	15 (9.2%)
2	63 (25%)	1 (0.6%)
> 2	47 (18%)	0 (0%)
Unknown	13	1
**Color blind**		
No	258 (97%)	160 (98%)
Yes	7 (2.6%)	1 (0.6%)
Don’t know	2 (0.7%)	2 (1.2%)
Unknown	2	1

Data reported as median (Q1, Q3) or n (%). ND: not determined; MSF_SC_: midpoint of sleep on free days corrected for sleep debt, determined using the μMCTQ.

* Values missing from two participants.

### Post-illumination pupillary response does not depend on time of day, age, or sex

**Figure 3:**
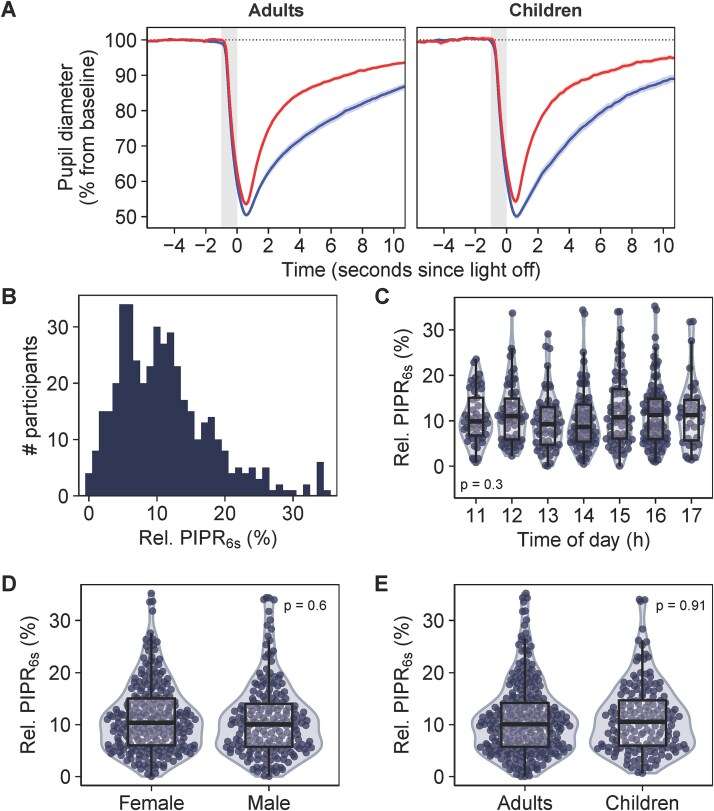
Effect of time of day, sex, and age on the PIPR. (A) Average pupil diameter time course in response to red (upper lines) and blue (lower lines) light in adults (*n* = 269) and children (*n* = 164). Data shown as mean and 95% CI. The dark gray boxes indicate the 1 s light stimulus. Light gray boxes indicate the time window used to calculate the PIPR_6s_. (B) Distribution of relative PIPR_6s_ across all participants (*n* = 433). (C) PIPR_6s_ by time of day at which the pupil recording was performed across all participants (*n* = 433). Time of day was binned per hour (e.g. pupil recordings that started between 10:30 and 11:30 are categorized as 11). *p*-value indicates the significance of time of day on PIPR_6s_ based on one-way ANOVA. (D) PIPR_6s_ by sex (*n* = 432). One participant who reported their sex as “other” was excluded from this panel. (E) PIPR_6s_ in adults and children (*n* = 433). *p*-values in panel C and D indicate the significance of sex and age on PIPR_6s_ based on student’s t-tests.

Absolute baseline pupil diameters prior to the red and blue light stimuli were highly correlated (r^2^ = 0.96), with slightly lower baseline values before the blue light stimulus than before the red light stimulus (Supplementary Figure S4). As expected, pupil dilation following light offset was slower in response to the 1-second blue light stimulus than to the 1-second red light stimulus (Figure 3A), resulting in an average PIPR_6s_ of 11.1 ± 0.3% (mean ± SEM), with a large degree of interindividual variability (Figure 3B). No evidence was found for an effect of time of day on PIPR_6s_ (F[6, 426] = 1.21, *p*=.301, ANOVA; Figure 3C), nor of sex on PIPR_6s_ (t[400] = 0.530, *p*=.596, t-test, Figure 3D). In addition, no evidence was found that PIPR_6s_ differed between adults and children (t[360] = –0.12, *p*=.905, t-test, Figure 3E).

### Sex- and age-specific relationship between chronotype and PIPR in adults

In adults, no significant crude association was found between chronotype and PIPR_6s_ (Supplementary Figure S5, left panel). However, in the full model testing the three-way and two-way interactions between PIPR_6s_, sex, and age on chronotype, we observed significant two-way interactions between PIPR_6s_ and age and between PIPR_6s_ and sex on chronotype, while the three-way interaction and the two-way interaction between sex and age were not significant (Table 2). This led to a final model that contains PIPR_6s_, age, and sex as main effects and the interaction terms between PIPR_6s_ and sex as well as PIPR_6s_ and age (Table 2). Furthermore, as an additional robustness check, we confirmed that both interactions remain significant when tested separately (Supplementary Table S1).

**Table 2:** Effects of PIPR, sex, age, and their interaction on chronotype in adults (*n* = 269)

Variables	Full model		Final model	
	F (df)^*^	*p*-value^*^	F (df)^*^	*p*-value^*^
PIPR_6s_	1.24 (1, 261)	0.266	1.29 (1, 263)	.258
Sex	9.33 (1, 261)	0.002	9.38 (1, 263)	.002
Age	20.2 (1, 261)	<0.001	20.3 (1, 263)	<.001
PIPR_6s_ x Sex	4.22 (1, 261)	0.041	4.53 (1, 263)	.034
PIPR_6s_ x Age	5.83 (1, 261)	0.016	5.92 (1, 263)	.016
Sex x Age	0.44 (1, 261)	0.507	—	—
PIPR_6s_ x Sex x Age	0.23 (1, 261)	0.631	—	—
	**Adjusted R** ^**2**^		**Adjusted R** ^**2**^	
Explained variance	0.111		0.116	

* F-statistics, degrees of freedom (df), and *p*-values obtained from type II sum-of-squares analysis of variance tests.

**Figure 4:**
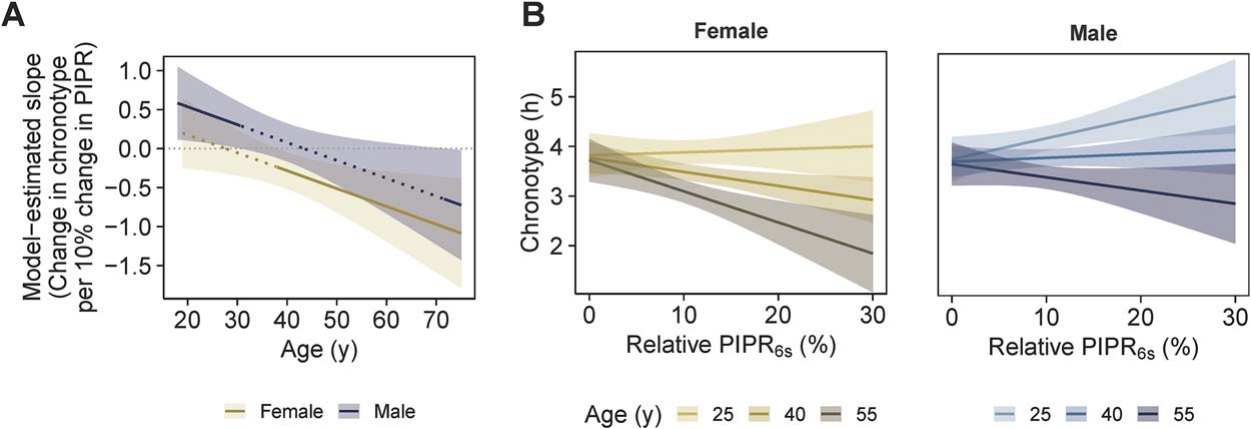
Model-estimated relationship between chronotype and PIPR_6s_ by sex and age in adults. (A) Model-estimated change in chronotype per 10% change in PIPR in males and females across the age range of the adult study population. Solid segments indicate that the slope is significantly different from 0 at the corresponding age, dotted segments indicate that the slope is not significantly different from 0 at the corresponding age. Shaded areas represent the 95% confidence intervals. (B) The model-estimated relationship between chronotype and PIPR_6s_ in adults at three representative ages (25, 40, and 55 years) in females (left) and males (right). These ages were selected to represent the age range of the adult study population (18–75 years) and do not reflect categorization of age in the statistical model. Lines and shaded areas in B represent estimated marginal means and 95% confidence intervals at representative ages derived from the multiple linear regression model predicting chronotype by interactions between PIPR_6s_ and sex and PIPR_6s_ and age (modeled as a continuous variable), i.e., the final model shown in Table 2.

Using the final model to interpret the direction and magnitude of the model terms (see Supplementary Table S2 for model coefficients), we find that the interaction between age and PIPR_6s_ is such that in young adults, a larger PIPR_6s_ is associated with a later chronotype. This relationship weakens with age and eventually reverses to a larger PIPR_6s_ being associated with an earlier chronotype in older participants (Figure 4A). The interaction between sex and PIPR_6s_ further shows that this age-dependent association differs by sex: across the entire age range, the model-estimated slope, representing the change in chronotype as a function of PIPR_6s_, is lower for women than for men: closer to 0 when the association was positive at younger ages, and more negative when the association reversed at older ages (Figure 4A). Using the Johnson–Neyman technique to further describe these interactions, we find that among women, PIPR_6s_ was significantly associated with chronotype at ages 38 years and older, such that larger PIPR_6s_ values predicted an earlier chronotype within this age range. In contrast, among men, a significant association of PIPR_6s_ and chronotype was observed at ages 31 years and younger, where larger PIPR_6s_ values were related to a later chronotype. Outside these age ranges, the relationship between PIPR_6s_ and chronotype was not statistically significant for either sex (Figure 4A). To facilitate interpretation of these findings, the model-estimated relationship between PIPR_6s_ and chronotype in men and women at three representative ages is visualized in Figure 4B.

Since our study, performed in a science museum, generated admittedly more noisy data compared to the highly-controlled laboratory studies that typically examine the relationship between pupillary responses and the circadian system, a sensitivity analysis on data from a more homogenous subset of participants was performed. When excluding data from participants that meet exclusion criteria commonly applied in controlled laboratory studies (see Methods for the complete description), the direction and magnitude of the interaction effects between sex and PIPR_6s_ on chronotype is similar to that observed in the entire study population (Supplementary Figure S6), further demonstrating the robustness of our findings.

### No relationship between chronotype and PIPR in children

In children, no overall significant relationship was found between chronotype and PIPR_6s_ (Supplementary Figure S5, right panel). Furthermore, no influence of age or sex on this relationship was present: we observed no significant three-way or two-way interactions between PIPR_6s_, sex, and age (Table 3; model coefficients in Supplementary Table S2). The direction and magnitude of the main effect of age on chronotype is such that the average 15-year old has a chronotype that is 1.54 [95% CI: 1.14–1.94] hours later than the average 9-year old (Supplementary Table S2), capturing the well-established shift towards later chronotypes during adolescence [41].

**Table 3:** Effects of PIPR, sex, age, and their interaction on chronotype in children (*n* = 163)^*^

Variables	Full model		Final model	
	**F (df)** ^#^	***p*-value** ^#^	**F (df)** ^#^	***p*-value** ^#^
PIPR_6s_	0.13 (1, 155)	.718	0.12 (1, 159)	.731
Sex	0.67 (1, 155)	.416	0.59 (1, 159)	.445
Age	55.8 (1, 155)	<.001	59.0 (1, 159)	<.001
PIPR_6s_ x Sex	0.03 (1, 155)	.87	—	—
PIPR_6s_ x Age	0.23 (1, 155)	.633	—	—
Sex x Age	0.09 (1, 155)	.762	—	—
PIPR_6s_ x Sex x Age	0.01 (1, 155)	.910	—	—
	**Adjusted R** ^**2**^		**Adjusted R** ^**2**^	
Explained variance	0.251		0.268	

^*^One participant reporting their sex as “Other” was excluded from this analysis;

^#^F-statistics, degrees of freedom (df), and *p*-values obtained from type II sum-of-squares analysis of variance tests.

## Discussion

Individual differences in chronotype arise from a complex combination of biological and environmental factors, including sex, age, and genetics. However, the extent to which sensitivity of the circadian system to light, mediated by melanopsin-mediated phototransduction, contributes to these differences remains unclear. Making use of the unique opportunity to address this question among a large and diverse sample of science museum visitors, we reveal a sex- and age-specific relationship between melanopsin-mediated light sensitivity and chronotype in adults, but not in children. Specifically, greater light sensitivity was associated with earlier chronotype in women in their late thirties and upwards, whereas it was associated with a later chronotype in young men (up to their early thirties), highlighting the important role of sex and age in shaping the relationship between light sensitivity and chronotype in adults. As such, this study provides novel insights into how light sensitivity interacts with age and sex to contribute to the variability of chronotype across the lifespan.

Without taking into account the effect of sex and age, no significant relationship between light sensitivity and chronotype was present in our data. We initially hypothesized that later chronotypes would have a larger PIPR, indicative of a circadian system that is more sensitive to light compared to earlier chronotypes. This hypothesis fits with other lines of evidence suggesting that individual differences in the sensitivity of the circadian clock to light are related to the timing of their circadian system. For example, Wright et al. [44] showed that in (predominantly male) participants who were exposed to only natural light (daylight and campfire) for a week, the circadian system synchronizes with the natural light–dark cycle. This effect was particularly large in individuals with late circadian timing (as measured by the dim light melatonin onset), who shifted to an earlier circadian timing when exposed to natural light–dark regimes. These data suggest that the habitual exposure, and potentially greater sensitivity to, evening artificial light is what (partially) leads to their late circadian timing. Contrary to our initial hypothesis, we find that the relationship between light sensitivity and circadian timing is more complex and depends on sex and age.

The finding that a larger PIPR is associated with an earlier chronotype in women but not in men could mechanistically be linked to previously identified sex differences in circadian function [11], such as differences in intrinsic circadian period [45] and light sensitivity of the circadian system [46]. Duffy et al. [45] found that the circadian period of women is six minutes shorter compared to men, which can account for differences in circadian timing between the sexes. Furthermore, Vidafar et al. [46] found that women exhibit a larger melatonin suppression than men under bright light (≥ 400 photopic lux) but not under moderate or dim light conditions (between 10 and 200 photopic lux). Consequently, the authors hypothesized that women might be more sensitive to the effects of bright (day)light on the circadian system than men. Together, a shorter circadian period and increased sensitivity to bright light suggest that women might be more sensitive to circadian phase advances than to phase delays, resulting in earlier circadian timing (e.g. chronotype) in more sensitive individuals, which is corroborated by our results. Future studies are necessary to validate the sex differences in circadian phase shifting and its resulting consequences on chronotype variation between the sexes.

Our study also adds to the existing evidence that the non-visual effects of light depend on age [10]. We find that age, in addition to sex, modulates the relationship between PIPR and chronotype in adults. Strikingly, when considering the combined additive effects of age and sex, we observe a more negative relationship between PIPR in women with increasing age, whereas young adult men have a more positive relationship between PIPR and chronotype that attenuates with increasing age. This effect of age may be (partly) explained by behavioral patterns of light exposure that differ across the lifespan [47]. Since our study took place in Amsterdam, we speculate that our adult study population consisted predominantly of parents of (young) children from near Amsterdam, where over 80% of school-aged children go to school on foot or by bike, often accompanied by their parents [48]. Thus, the many adults aged in their 40s in our sample are likely habitually exposed to substantial dose of high-intensity daylight in the morning for a large part of the year. Mathematical models of the circadian system show that brighter daytime light conditions and decreased evening light exposure lead to earlier circadian phase and sleep timing [49–51]. Therefore, individuals with a higher light sensitivity who are exposed to light in the morning may have earlier circadian timing, and, consequently, an earlier chronotype [9]. These results emphasize the highly nuanced interactions between underlying physiology (e.g. in circadian period or light sensitivity) and behavioral light exposure, which should be considered in future investigations.

Although the magnitude of the PIPR itself did not differ between children and adults in our study, consistent with previous findings [52], the interaction of PIPR with age and sex that we observed in adults was not present in children. This discrepancy suggests developmental differences in the relationship between melanopsin-mediated phototransduction and chronotype, a topic that has received limited attention. Due to the enrichment for primary school-aged children and underrepresentation of pubescent and adolescent children in our sample, a potential differential effect of sex emerging during puberty might be absent in our data. Since sex differences, which are amplified during puberty due to an increase in circulating sex hormones, may influence circadian physiology [53], pre-pubertal children might exhibit a different relationship between PIPR and chronotype than adolescents and adults. Therefore, further research stratified by age, specifically in children and adolescents, is required to further examine the developmental changes in PIPR dynamics and light sensitivity of the circadian system during childhood and adolescence [54, 55].

A strength of this study was its unique setting in a science museum, which allowed us to recruit a large and diverse sample in a relatively short time period. Importantly, the large number of participants willing to participate in our study during their museum visit emphasizes the potential of using such a setting to promote awareness of circadian biology and the physiological effects of light in the wider community, while simultaneously collecting valuable scientific data. A limitation of this setting is that our study was relatively uncontrolled and designed to take up as little time as possible from the participants’ perspective so that it easily fitted within their museum visit. This limited time frame is reflected in several aspects of our study design: the light stimuli were presented to the participants only once, in a fixed order (red followed by blue; to keep the interstimulus interval short), and not repeated in case of blinks during light exposure or other artifacts; the pupillary recordings was optimized to determine the PIPR_6s_ rather than more sustained PIPR measures; the μMCTQ was used as a quick but validated continuous measure of chronotype [23]; and we were unable to determine relationships with other related constructs such as diurnal preference using the morningness-eveningness questionnaire [56]. In addition, the study population was substantially more heterogeneous than the controlled laboratory studies that are typically conducted in the field of chronobiology to study light sensitivity or circadian timing. While this heterogeneity increases the ecological validity of our findings, it may have introduced noise or confounders. However, the sensitivity analysis on a more homogenous subset of participants showed the same direction of the sex and age effects, supporting the robustness of our findings. Furthermore, our study was conducted during a two-week timeframe near the winter solstice, during which the availability of daylight in Amsterdam is limited (approximately 7 hours and 42 minutes). Although we consider this a strength of our study, since all participants were exposed to a similar (outdoor) photoperiod, it remains to be investigated to what extent our findings apply in other seasons, especially since season was found to be an important determinant of the relationship between PIPR and circadian timing [20]. Given that our observed interactions may be partially driven by differences in habitual light exposure, it will be particularly interesting to investigate the degree to which they persist or change under changing day lengths across the year and depend on intra-individual differences in prior light history.

In summary, we show that sex and age influence the relationship between melanopsin-mediated phototransduction and chronotype, providing novel evidence on the highly dynamic interplay between the sensitivity of the circadian clock to light and chronotype. It is striking that a greater sensitivity to light has the opposite relationship with chronotype in young adult men compared to older adult women. This suggests that individual variation in light sensitivity interacts with other factors, such as sex- and age-specific differences in behavior or circadian biology, to influence circadian timing. Altogether, our findings call for further research into the effect of sex, age, and daily light exposure patterns, on the relationship between light sensitivity and circadian timing, and may be used for the development of individualized light exposure recommendations.

## Supplementary Material

supp-info_final_zsag057

## Data Availability

Data and code generated in this study are available on Zenodo (https://doi.org/10.5281/zenodo.16810838).
