# Supplementary material for: Sex and age modulate the relationship between melanopsin-dependent light sensitivity and chronotype
Source: Sleep. 2026 Feb 27;49(6):zsag057. doi: 10.1093/sleep/zsag057 (PMC13267903; doi:10.1093/sleep/zsag057)
Supplement: supp-info_final_zsag057 [file supp-info_final_zsag057.pdf]

# – Supplementary Information –

Sex and age modulate the relationship between melanopsin-dependent light sensitivity and chronotype

van der Zwet G[1,*], Bor Z[1,*], Bos R[1,2], van Dorp R[3], Pape LM[4], van der Zwet LCA[3], van Dijk EHC[5,6], van de Stadt H[7], McGlashan EM[8], Michel S[1,3], Kervezee L[1,#]

1. Group of Circadian Medicine, Department of Cell and Chemical Biology, Leiden University Medical Center, Einthovenweg 20, 2333 ZC, Leiden, The Netherlands
2. Department of Neonatology, Wilhelmina Children's Hospital, University Medical Center Utrecht, Heidelberglaan 100, 3584 CX, Utrecht, Netherlands
3. Group of Neurophysiology, Department of Cell and Chemical Biology, Leiden University Medical Center, Einthovenweg 20, 2333 ZC, Leiden, The Netherlands
4. Department of Clinical Psychology, Faculty of Social and Behavioural Sciences, Leiden University, Wassenaarseweg 52, 2333 AK, Leiden, The Netherlands
5. Department of Ophthalmology, Leiden University Medical Center, Albinusdreef 2, 2333 ZA Leiden, The Netherlands
6. Department of Vitreoretinal Surgery, Rotterdam Eye Hospital, Rotterdam, The Netherlands
7. Department of Medical Technology, Leiden University Medical Center, Einthovenweg 20, 2333 ZC, Leiden, The Netherlands
8. Melbourne School of Psychological Sciences, University of Melbourne, Parkville, Victoria, 3010, Australia

[*] These authors contributed equally and share first authorship

[#] To whom correspondence should be addressed: L.Kervezee@lumc.nl

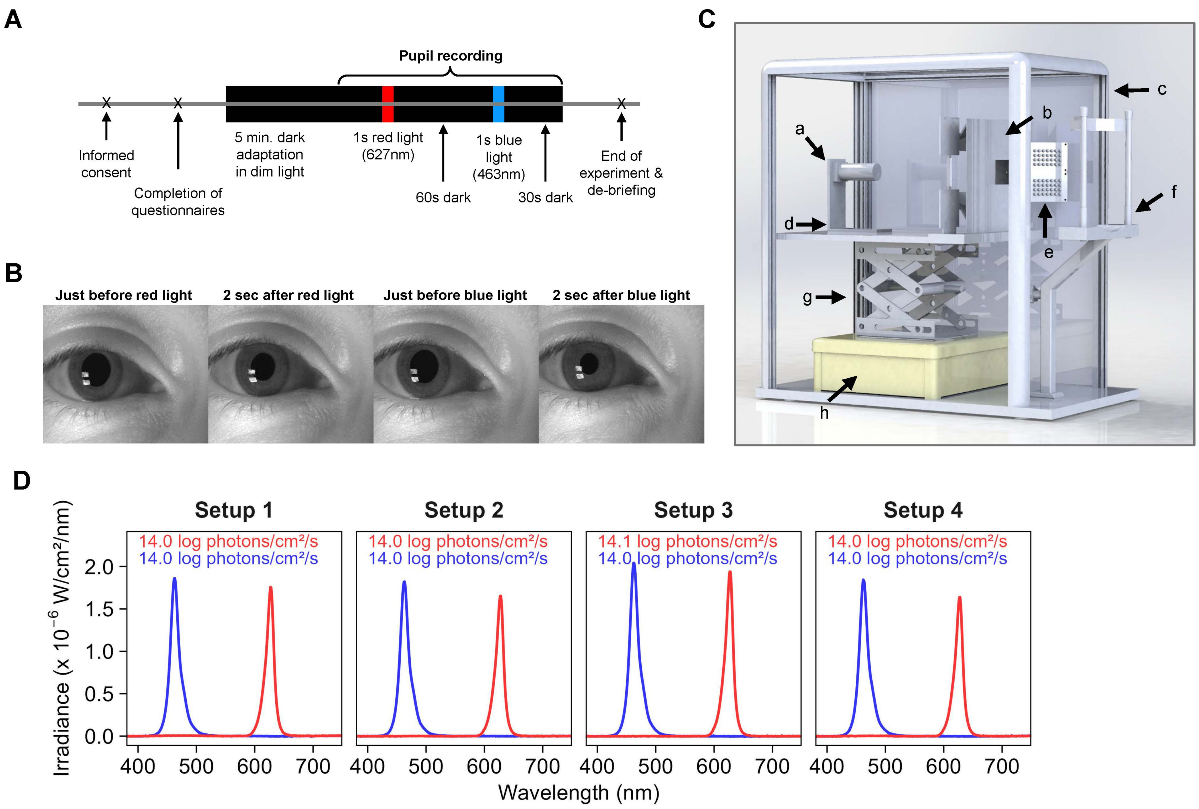

Supplementary Figure S1. Study design. (A) Overview of the experimental protocol. **(B)** Example of the personalized images shown to participants as feedback at the end of the experimental session. **(C)** Three-dimensional rendering of the experimental setup with labels indicating the different components. a: monochrome camera; b: light panel (RGB LEDs covered with diffuser material); c: casing around the setup; d: horizontal adjustable platform; e: infrared light source; f: headrest; g: vertical adjustable platform; h: casing containing controller. **(D)** Spectral irradiance distribution of the red and blue light stimuli generated by each of the four experimental setups. Text insets show total irradiance per setup for the red (top) and blue (bottom) light stimuli.

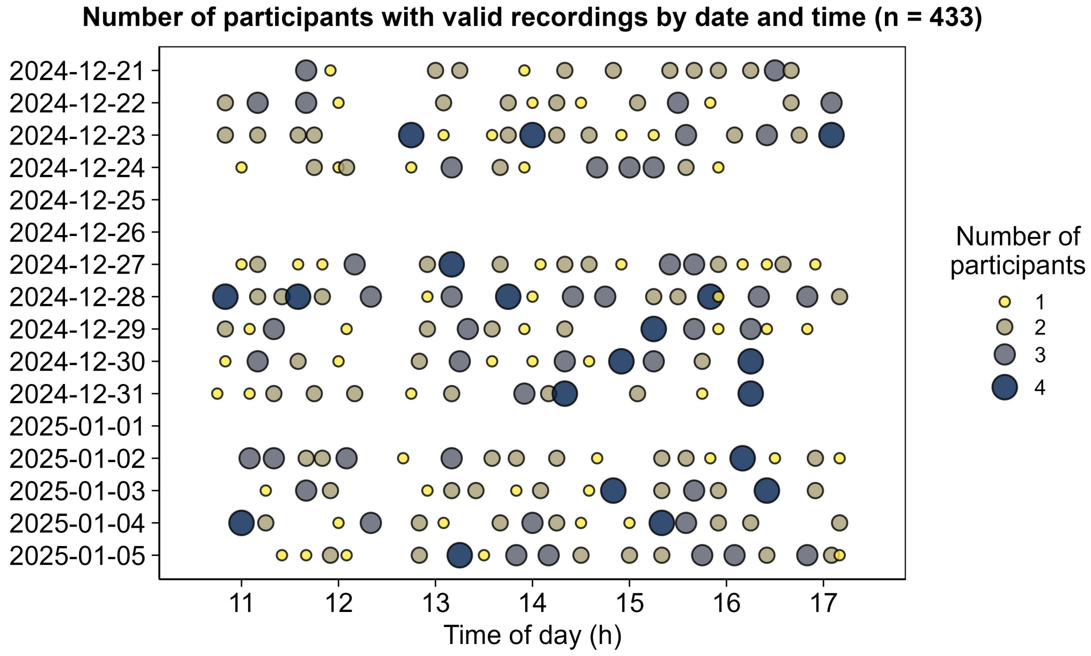

Supplementary Figure S2. Number of participants by date and time. The experimental setup allowed for measuring up to four individuals simultaneously, resulting in the measurement of one to four participants per session.

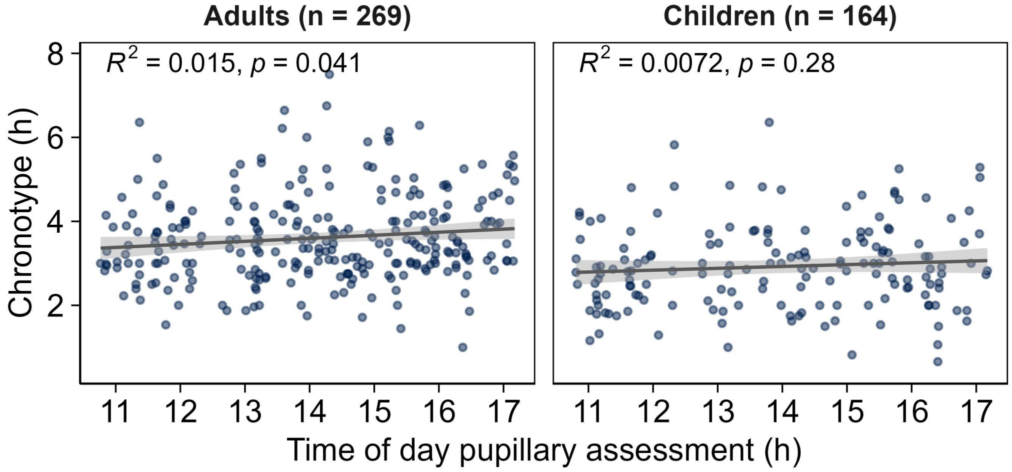

Supplementary Figure S3. Relationship between the time of day that participants underwent the pupillary assessment and chronotype. Analysis is split by adults (left panel) and children (right panel). The grey line and shaded area represents the regression line with the standard error; the accompanying p-values and r^2 values are shown in the panel.

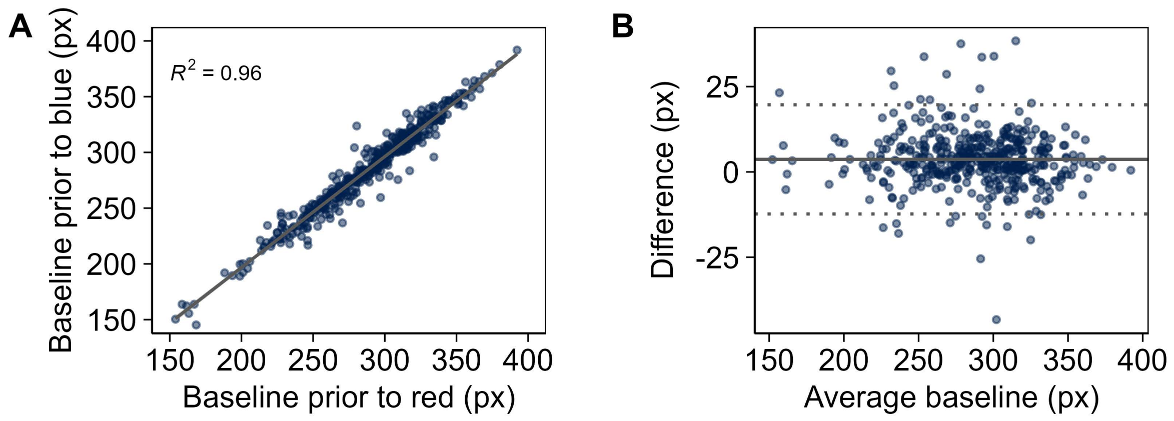

Supplementary Figure S4. Comparison of baseline pupil diameter prior to the red and blue light stimuli. (A) Relationship between baseline pupil diameter before the red light pulse and the baseline pupil diameter before the blue light pulse in all participants (n = 433). Pupil diameters are shown in pixels. The solid line represents the linear regression line through the data points. The corresponding R^2 value is shown in the plot. **(B)** Bland-Altman plot of the average of the baseline pupil diameter (in pixels) prior to the red and blue light pulses against the difference between the baseline pupil diameters before the red and the blue light pulses in all participants. The solid line represents the average difference in baseline, the dashed lines indicate 95% limits of agreement.

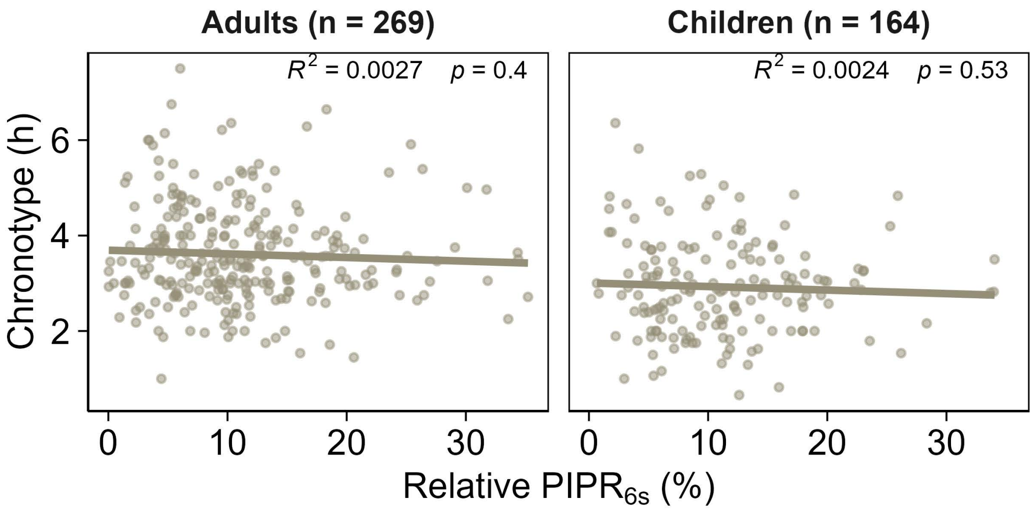

Supplementary Figure S5. The crude association between chronotype and the relative post-illumination pupillary response (PIPR) at 6 seconds after the light offset in the raw data. The analysis is separated for children and adults. The dots represent the individuals. The line represents the correlation between chronotype and relative $PIPR_{6s}$. The corresponding p values and R^2 values are shown in the plots. This analysis does not account for the effect of sex and age.

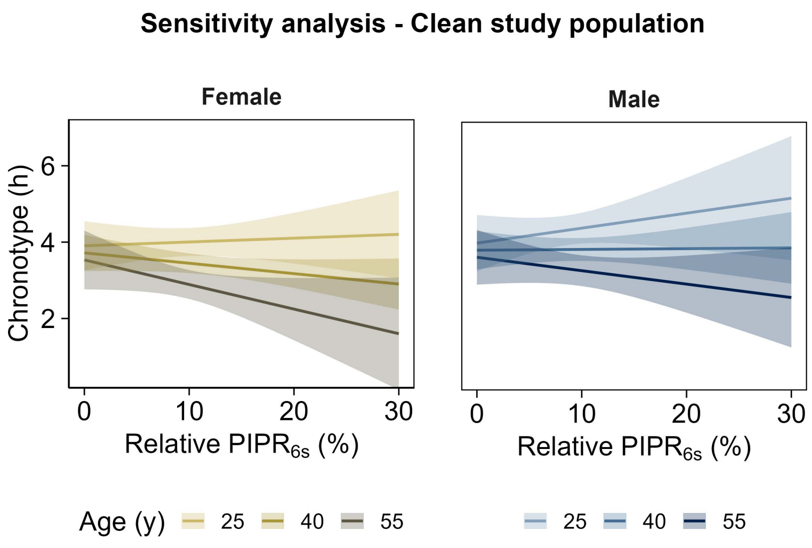

Supplementary Figure S6. Sensitivity analysis. Model-estimated relationships between the post-illumination pupillary response $(PIPR)_{6s}$ and chronotype at three representative ages in adult males and females in a 'clean' study population (see Methods for a full description of this subset). In total, 116 participants (out of the 269 adult participants) were included in this analysis (68 women, 48 men). Although the interaction effects are no longer significant ($PIPR_{6s}$ x Sex: $F(1, 110) = 1.06$, $p = 0.305$; $PIPR_{6s}$ x Age: $F(1, 110) = 1.99$, $p = 0.161$), the direction and magnitude of the effects are similar to that of the main analysis (see Figure 4B in the main text).

Supplementary Table S1. Effects of post-illumination pupillary response (PIPR), sex, age, and their interaction when tested separately on chronotype in adults (n = 269).

**Variables**	**$PIPR_{6s}$ x Sex interaction**		**$PIPR_{6s}$ x Age interaction**	
	**F (df)#**	**P-value#**	**F (df)#**	**P-value#**
$PIPR_{6s}$	1.26 (1, 264)	0.262	1.27 (1, 264)	0.261
Sex	9.15 (1, 264)	0.003	9.25 (1, 264)	0.003
Age	19.9 (1, 264)	<0.001	19.9 (1, 264)	<0.001
$PIPR_{6s}$ x Sex	4.50 (1, 264)	0.035	-	-
$PIPR_{6s}$ x Age	-	-	5.91 (1, 264)	0.016
	**Adjusted R^2**		**Adjusted R^2**	
Explained variance	0.099		0.104	

Supplementary Table S2. Model coefficients of the final models fitted on data from adults and children to predict chronotype. Coefficients of continuous variables are centered and reported on their original (not standardized) scale for ease of interpretation.

Model term	Adults (n = 269)		Children (n = 163)	
	Coefficient	SE	Coefficient	SE
Intercept	3.4	0.08	3.0	0.09
10*$PIPR_{6s}$	-0.30	0.12	-0.035	0.10
Sex [ref: Female]	0.37	0.12	-0.10	0.14
Age	-0.029	0.0058	0.257	0.033
10*$PIPR_{6s}$ x Sex	0.36	0.17	-	-
10*$PIPR_{6s}$ x Age	-0.023	0.009	-	-
